# Supplementary material for: Dynamic tracking and identification of tissue-specific secretory proteins in the circulation of live mice
Source: Nat Commun. 2021 Sep 1;12:5204. doi: 10.1038/s41467-021-25546-y (PMC8410947; doi:10.1038/s41467-021-25546-y)
Supplement: Supplementary file 1 — Supplementary information [file 41467_2021_25546_MOESM1_ESM.pdf]

Supplementary Information

Dynamic tracking and identification of tissue-specific secretory proteins in the circulation of live mice

Authors

Kwang-eun Kim, Isaac Park, Jeesoo Kim, Myeong-Gyun Kang, Won Gun Choi, Hyemi Shin, Jong-Seo Kim, Hyun-Woo Rhee and Jae Myoung Suh

Kim et al., Supplementary Figure 1

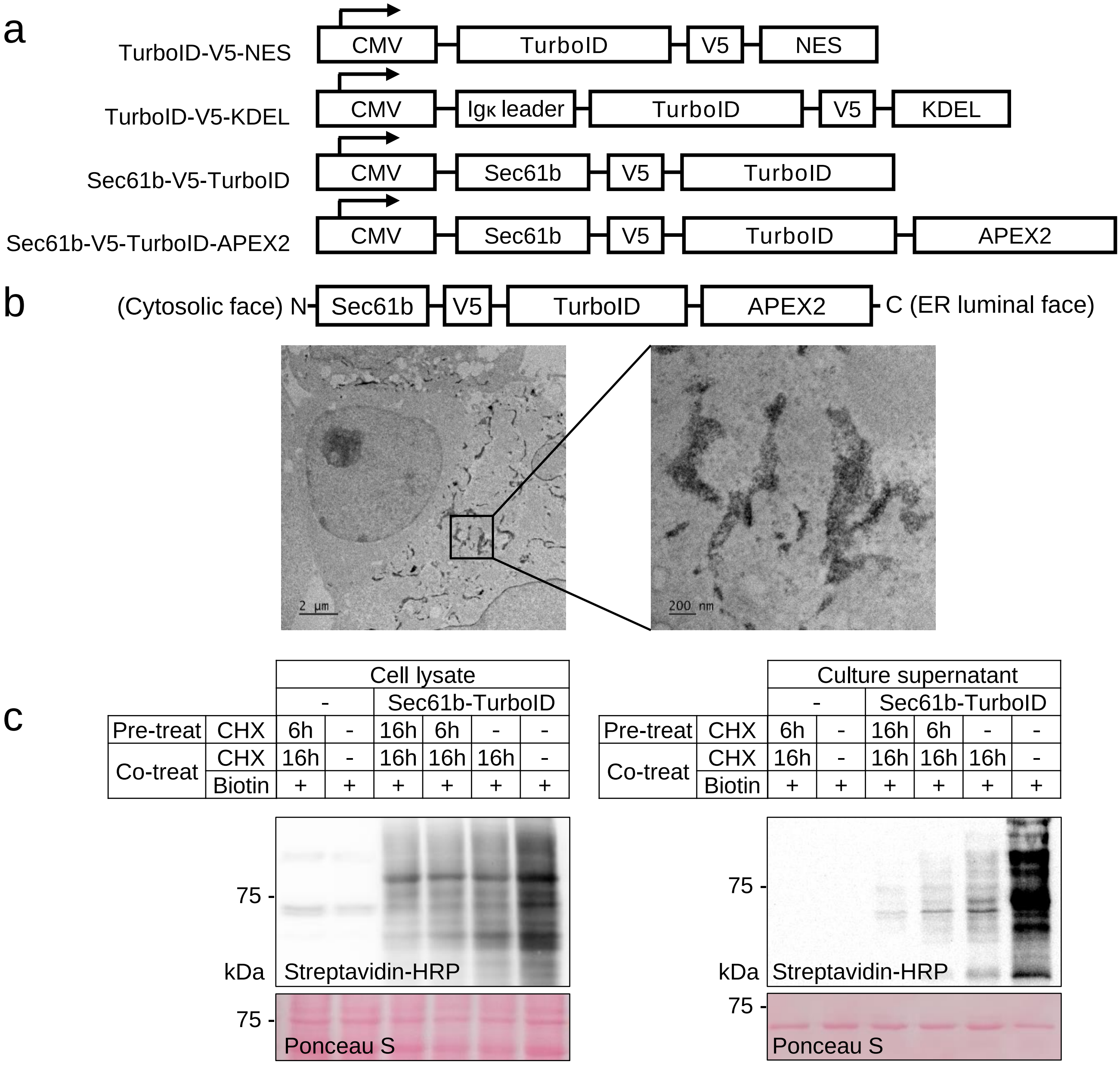

Supplementary Fig. 1 Schematic of constructs used in this study and validation of Sec61b-TurboID construct. a, Schematic of constructs used in this study. CMV, human cytomegalovirus promoter; Igκ, immunoglobulin κ; NES, nuclear export signal; APEX2, engineered ascorbate peroxidase. **b,** Transmission electron microscope images of cells expressing Sec61b-TurboID-APEX2 targeted to the ER lumen. HEK293 cells were transfected with Sec61b-TurboID-APEX2 expression vector. Dark regions indicate the presence of peroxidase staining by Sec61b-TurboID-APEX2 reacting with diaminobenzidine (DAB) and H_2O_2. Scale bars, 2μm and 200nm. These experiments were repeated as biological triplicates with similar results. **c**, Effect of Cycloheximide (CHX, 50 μg/ml) on biotin-labeled proteins in cell lysates or culture supernatants of HEK293 cells expressing Sec61b-V5-TurboID. These experiments were repeated as biological triplicates with similar results.

Kim et al., Supplementary Figure 2

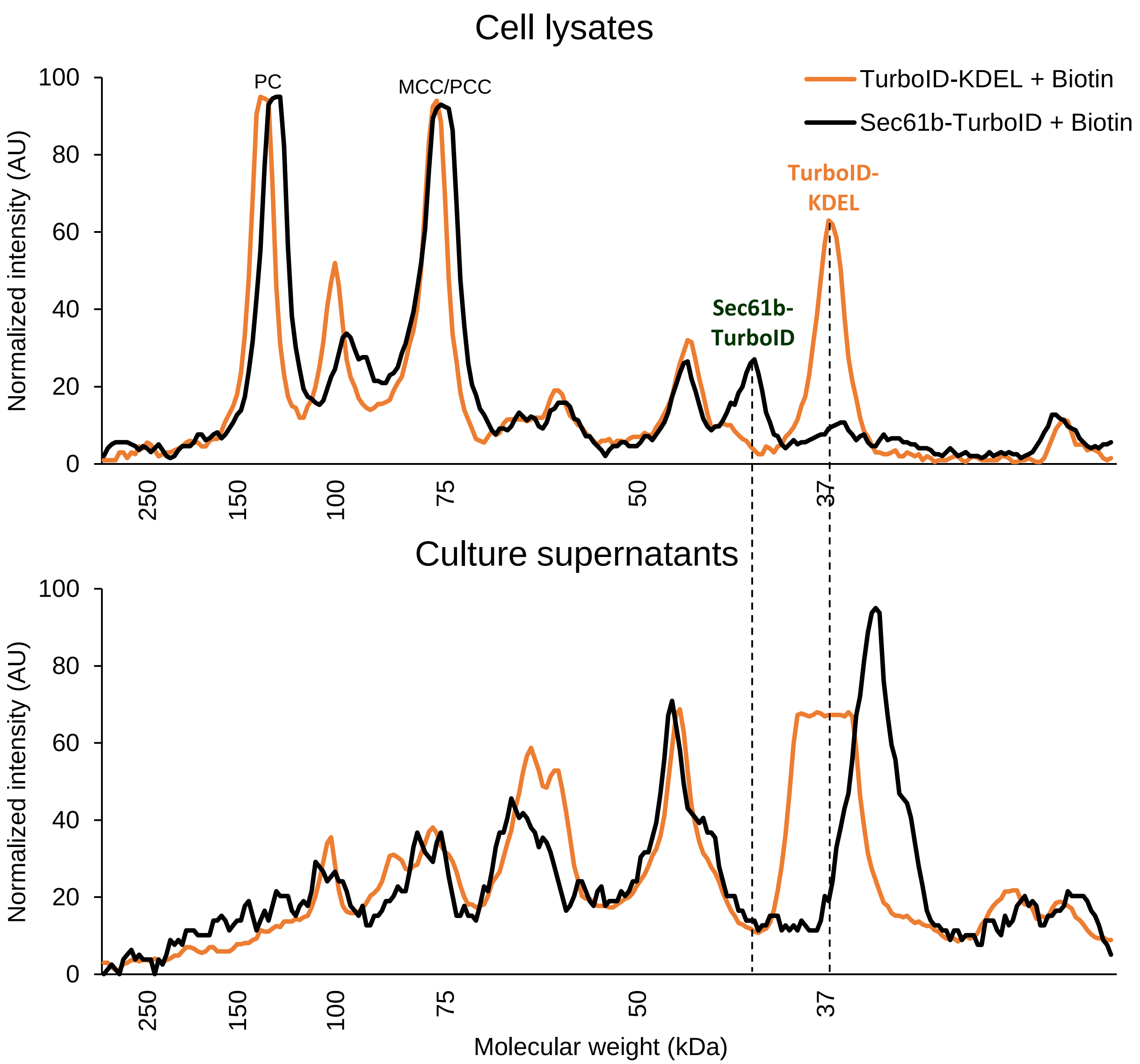

Supplementary Fig. 2 Biotin-labeled protein profiles of cell lysates and culture supernatants from TurboID-KDEL and Sec61b-TurboID expressing cells. Line-scan analysis of biotin-labeled proteins detected by streptavidin-HRP blots in cell lysates and culture supernatants of NIH-3T3 cells expressing TurboID-KDEL (orange) or Sec61b-TurboID (black) and treated with biotin. PC, Pyruvate carboxylase; MCC/PCC, Methylcrotonyl-CoA carboxylase/Propionyl-CoA carboxylase. Source data are provided as a Source Data file.

Kim et al., Supplementary Figure 3

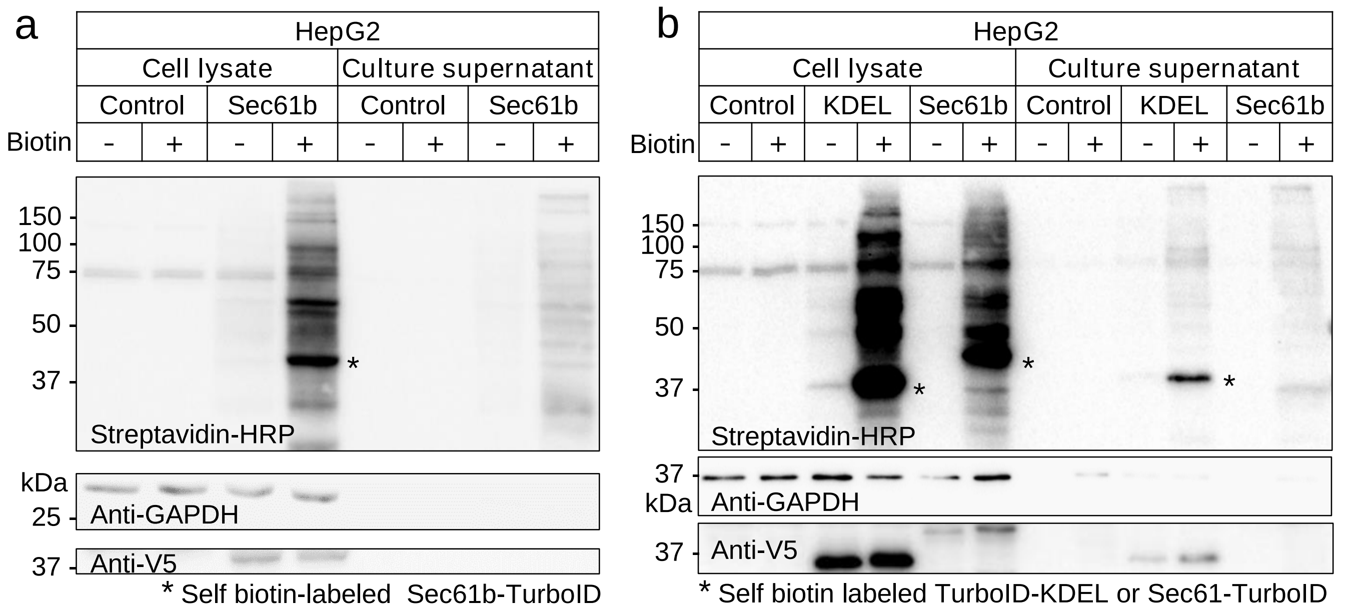

Supplementary Fig. 3 Self-secretion of TurboID-KDEL in HepG2 cells. a, Western blots for biotin-labeled proteins (Streptavidin-HRP) and Sec61b-TurboID (Anti-V5) in cell lysates or culture supernatants of HepG2 cells expressing GFP (Control) or Sec61b-TurboID (Sec61b). These experiments were repeated as biological triplicates with similar results. Source data are provided as a Source Data file. **b**, Western blots for biotin-labeled proteins (Streptavidin-HRP) and TurboID (Anti-V5) in cell lysates or culture supernatants of HepG2 expressing GFP (Control), TurboID-KDEL (KDEL) or Sec61b-TurboID (Sec61b). Anti-GAPDH is a loading control. Asterisks indicate self biotin-labeled TurboID-KDEL or Sec61-TurboID. These experiments were repeated as biological triplicates with similar results. Source data are provided as a Source Data file.

Kim et al., Supplementary Figure 4

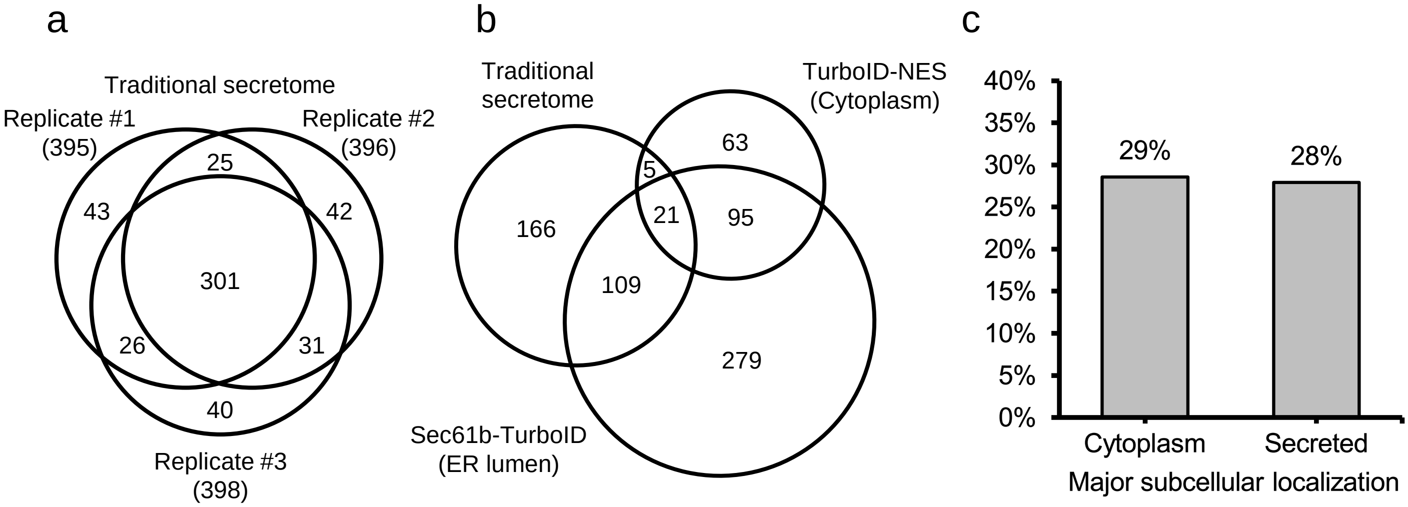

Supplementary Fig. 4 Traditional secretome analysis. a, Venn diagram depicting proteins with more than two spectral counts for each biological replicate in culture supernatant of control HEK293 cells. **b,** Venn diagram depicting traditional secretome analysis of culture supernatant from HEK293 cells and biotinylated protein analysis of culture supernatant from HEK293 cells expressing TurboID-NES or Sec61b-TurboID. **c,** Fraction of cytoplasmic and secreted proteins from traditional secretome analysis. Source data are provided as a Source Data file.

Kim et al., Supplementary Figure 5

a

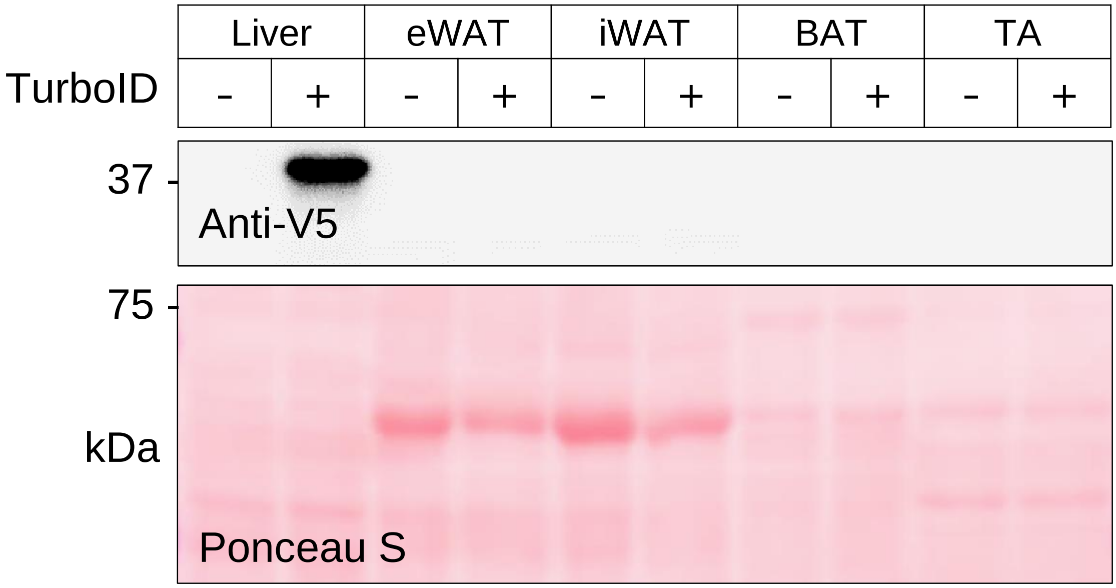

b

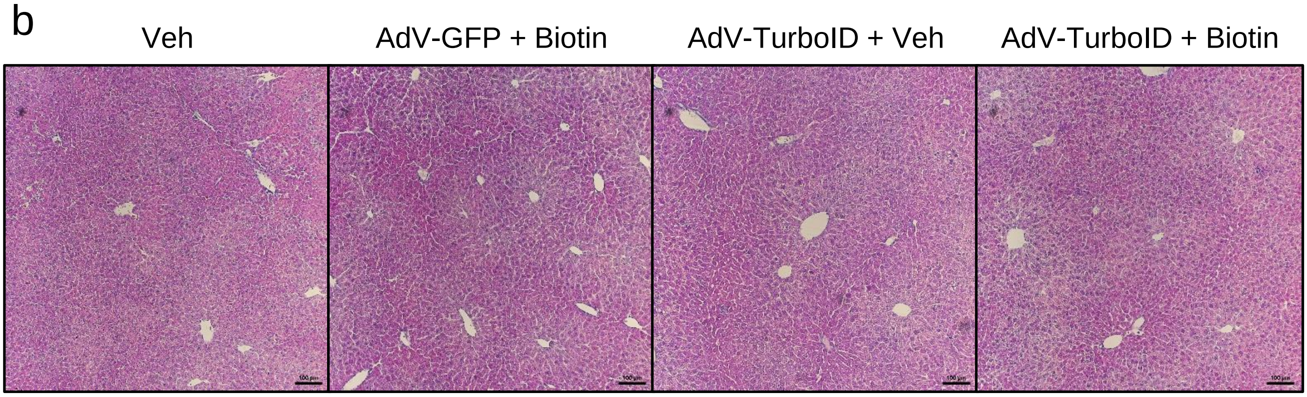

Supplementary Fig. 5 Validation of liver-specific Sec61-TurboID expression and liver histology. a, Western blot for Sec61b-TurboID (Anti-V5) in liver and other tissues. eWAT, epididymal white adipose tissue; iWAT, inguinal white adipose tissue; BAT, brown adipose tissue; TA, tibialis anterior. These experiments were repeated as biological triplicates with similar results. Source data are provided as a Source Data file. **b**, Hematoxylin and Eosin staining of liver tissues from mice with indicated treatment. Vehicle (Veh), GFP expressing adenovirus (AdV-GFP), Sec61b-TurboID expressing adenovirus (AdV-TurboID). Scale bars, 100 μm. These experiments were repeated as biological triplicates with similar results.

Kim et al., Supplementary Figure 6

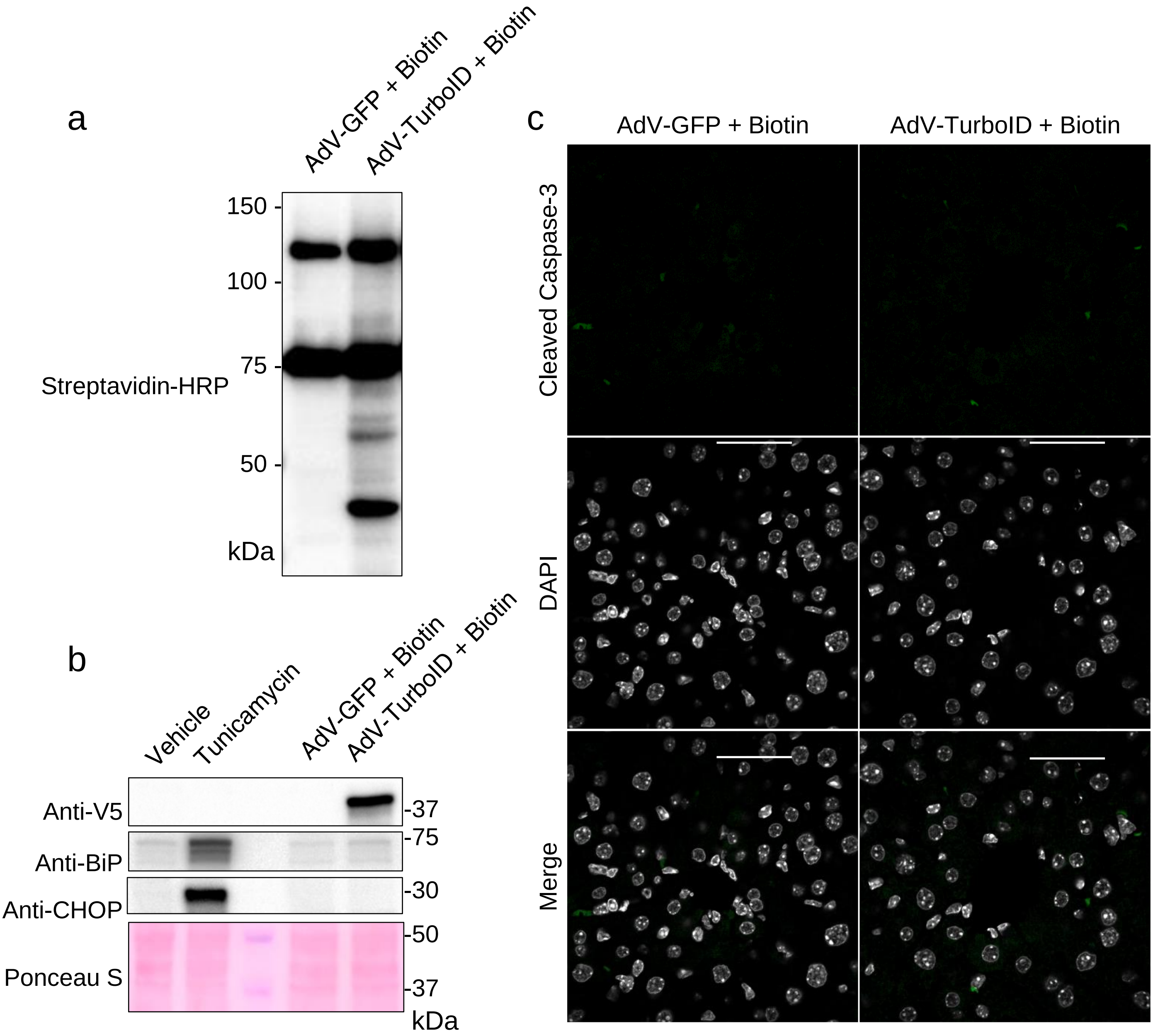

Supplementary Fig. 6 Analysis of ER stress and apoptosis markers in liver tissue of mice transduced with Sec61b-TurboID adenovirus. a, Western blots for biotin-labeled proteins (Streptavidin-HRP) in liver lysates from mice transduced with GFP or Sec61b-TurboID adenovirus. These experiments were repeated as biological triplicates with similar results. Source data are provided as a Source Data file. **b**, Western blots for Sec61b-TurboID (Anti-V5), BiP, and CHOP in liver lysates from mice transduced with GFP or Sec61b-TurboID adenovirus. For positive control, tunicamycin (1 mg/kg) was injected to mice and liver was harvested 16h after injection. These experiments were repeated as biological triplicates with similar results. Source data are provided as a Source Data file. **c**, Immunofluorescence imaging of cleaved caspase-3 and DAPI in liver sections from mice transduced with GFP or Sec61b-TurboID adenovirus. Scale bars, 50 μm. These experiments were repeated as biological triplicates with similar results.

Kim et al., Supplementary Figure 7

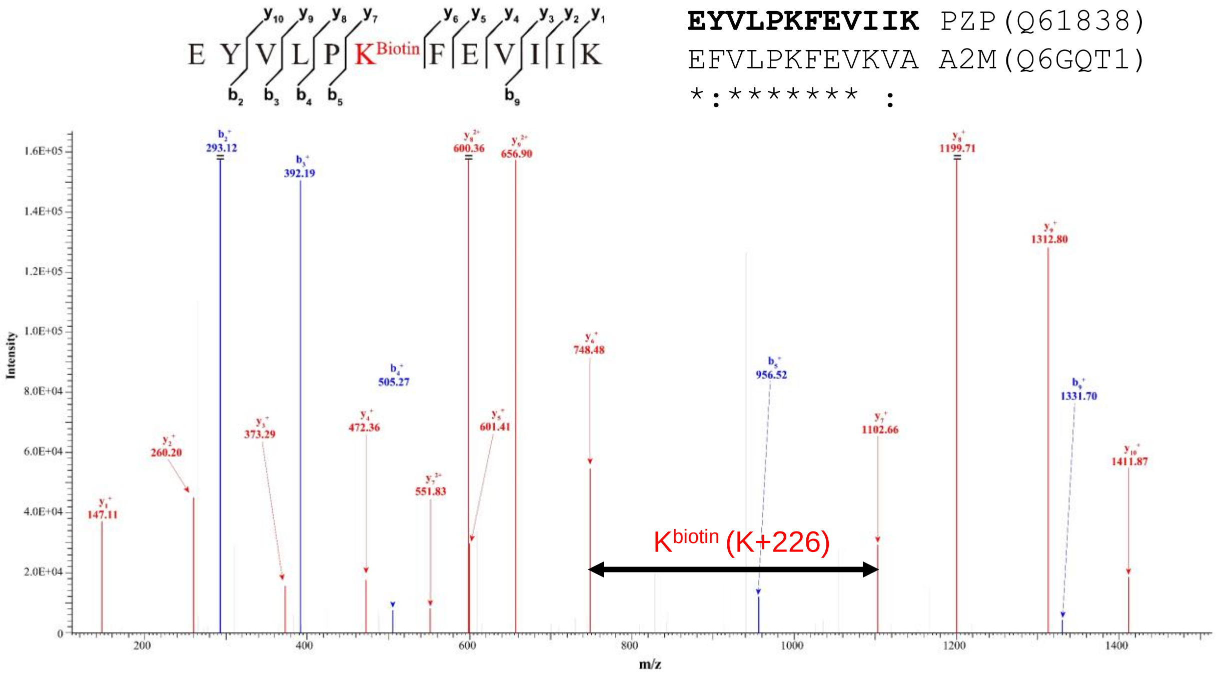

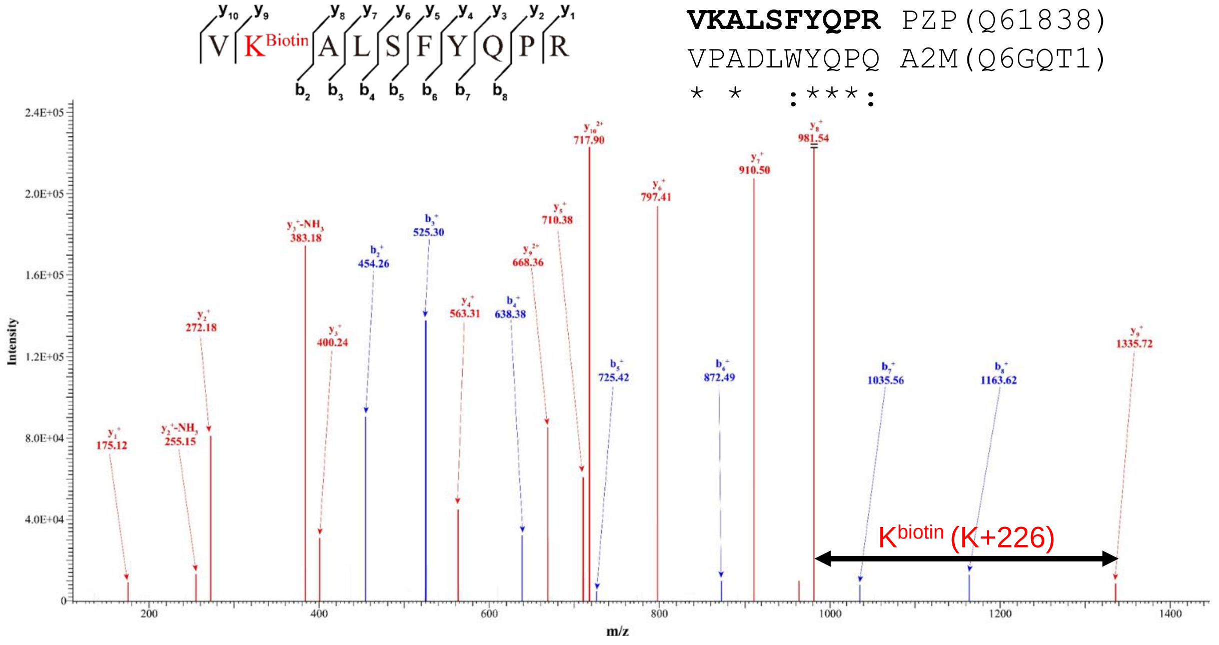

Supplementary Fig. 7 Representative tandem mass spectrum of biotinylated PZP peptides. Mass of the biotinylated lysine residue is 354 Da (K+226 Da). Arrow represents the mass shift of the biotinylated lysine residue from the biotinylated PZP peptide (Q61838).

Kim et al., Supplementary Figure 8

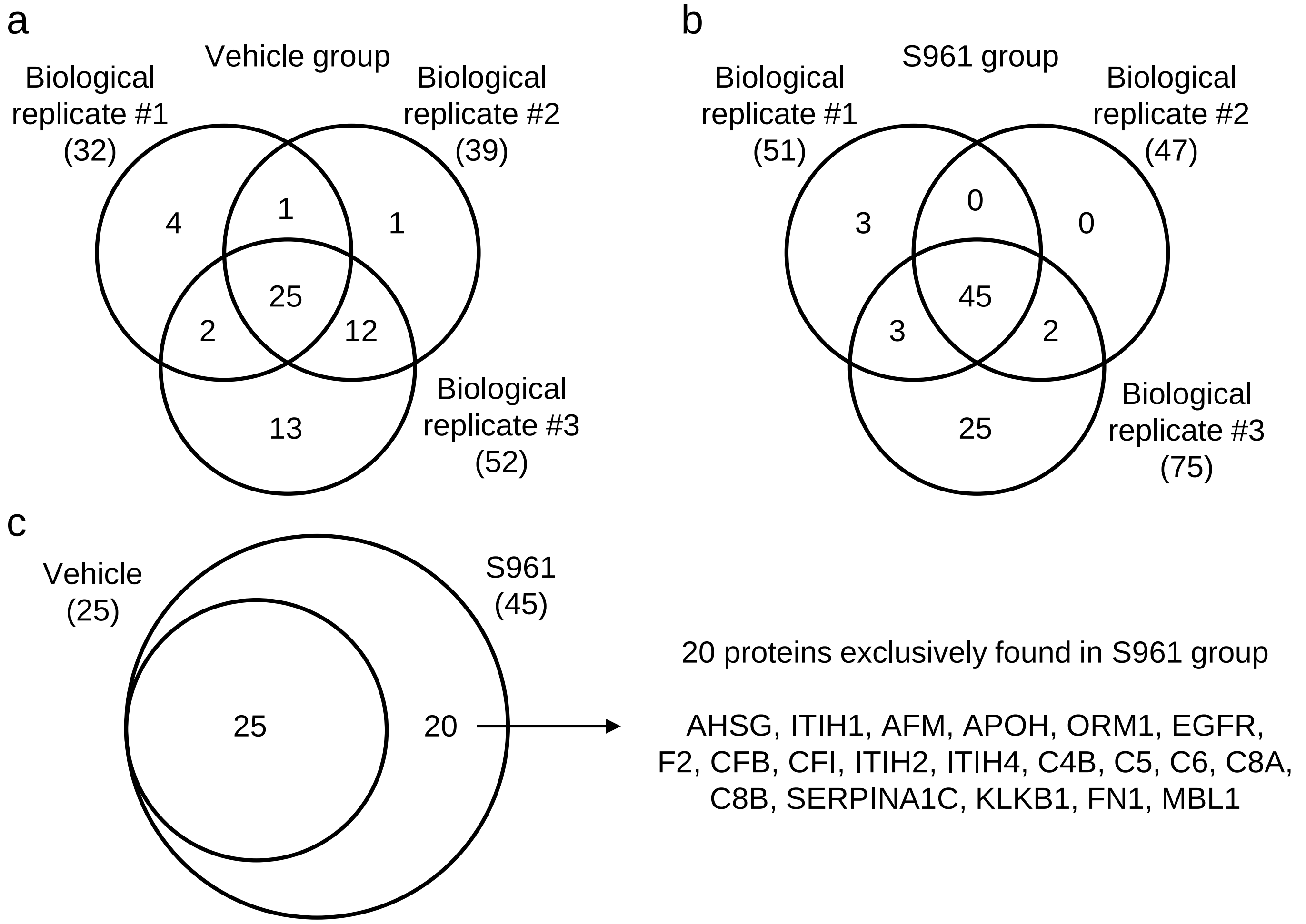

Supplementary Fig. 8 Venn diagrams depicting biotinylated secretory proteins identified in plasma of liver *i*SLET mice treated with vehicle or S961. a, **b** Venn diagrams depicting biotinylated proteins with more than two biotinylated spectral counts for each biological replicate in vehicle treated group (**a**) and in S961 treated group (**b**), respectively. **c**, Venn diagram depicting proteins identified in plasma of liver *i*SLET mice treated with vehicle or S961. AHSG, Alpha-2-HS-glycoprotein; ITIH1, Inter-alpha-trypsin inhibitor heavy chain H1; AFM, Afamin; APOH, Beta-2-glycoprotein 1; ORM1, Alpha-1-acid glycoprotein 1; EGFR, Receptor protein-tyrosine kinase; F2, Coagulation factor II; CFB, Complement factor B; CFI, Complement factor I; ITIH2, Inter-alpha-trypsin inhibitor heavy chain H2; ITIH4, Inter alpha-trypsin inhibitor, heavy chain 4; C4B, Complement C4-B; C5, Complement C5; C6, Complement component 6; C8A, Complement component C8 alpha chain; C8B, Complement component C8 beta chain; SERPINA1C, Alpha-1-antitrypsin 1-3; KLKB1, Plasma kallikrein; FN1, Fibronectin; MBL1, Mannose-binding protein A. Source data are provided as a Source Data file.
