# Supplementary material for: Dynamic tracking and identification of tissue-specific secretory proteins in the circulation of live mice
Source: Nat Commun. 2021 Sep 1;12:5204. doi: 10.1038/s41467-021-25546-y (PMC8410947; doi:10.1038/s41467-021-25546-y)
Supplement: Supplementary file 3 — Description of Additional Supplementary Files [file 41467_2021_25546_MOESM3_ESM.docx]

**Description of Additional Supplementary Files**

**File Name:** Supplementary Data 1

**Description:** Identified liver secretory proteins in mouse plasma with definitive in situ biotinylation evidence on lysine residues and comparison with other studies.

**File Name:** Supplementary Data 2

**Description:** Summary of identified peptide-to-spectrum (PSM) match list of biotinylated peptides in mouse plasma.

**File Name:** Supplementary Data 3

**Description:** Summary of plasmid information.
