# Supplementary material for: Dynamic tracking and identification of tissue-specific secretory proteins in the circulation of live mice
Source: Nat Commun. 2021 Sep 1;12:5204. doi: 10.1038/s41467-021-25546-y (PMC8410947; doi:10.1038/s41467-021-25546-y)
Supplement: Supplementary file 7 — Reporting Summary [file 41467_2021_25546_MOESM7_ESM.pdf]

nature research

Corresponding author(s): Jong-Seo Kim, Hyun-Woo Rhee, Jae Myoung Suh

Last updated by author(s): Aug 5, 2021

Reporting Summary

Nature Research wishes to improve the reproducibility of the work that we publish. This form provides structure for consistency and transparency in reporting. For further information on Nature Research policies, see our Editorial Policies and the Editorial Policy Checklist.

Statistics

For all statistical analyses, confirm that the following items are present in the figure legend, table legend, main text, or Methods section.

n/a	Confirmed	
☐	☒	The exact sample size (n) for each experimental group/condition, given as a discrete number and unit of measurement
☐	☒	A statement on whether measurements were taken from distinct samples or whether the same sample was measured repeatedly
☐	☒	The statistical test(s) used AND whether they are one- or two-sided<br>*Only common tests should be described solely by name; describe more complex techniques in the Methods section.*
☒	☐	A description of all covariates tested
☒	☐	A description of any assumptions or corrections, such as tests of normality and adjustment for multiple comparisons
☐	☒	A full description of the statistical parameters including central tendency (e.g. means) or other basic estimates (e.g. regression coefficient) AND variation (e.g. standard deviation) or associated estimates of uncertainty (e.g. confidence intervals)
☐	☒	For null hypothesis testing, the test statistic (e.g. F, t, r) with confidence intervals, effect sizes, degrees of freedom and P value noted<br>*Give P values as exact values whenever suitable.*
☒	☐	For Bayesian analysis, information on the choice of priors and Markov chain Monte Carlo settings
☒	☐	For hierarchical and complex designs, identification of the appropriate level for tests and full reporting of outcomes
☒	☐	Estimates of effect sizes (e.g. Cohen's d, Pearson's r), indicating how they were calculated

Our web collection on statistics for biologists contains articles on many of the points above.

Software and code

Policy information about availability of computer code

Data collection	ChemiDoc XRS+ System with Image Lab Software v.6.0.1 (Bio-Rad, 1708265) was used to acquire western blots imaged with chemiluminescent HRP substrate (Immobilon, P90720). Confocal Laser Scanning Microscopes (Leica, SP8X and Zeiss, LSM 780) with Leica Application Suite X v.3.7.1.21655 and ZEN blue v.3.1 software were used to acquire imaging data. All MS/MS datasets were first subject to peak picking and mass recalibration processed with RawConverter v.1.2.0.0 (http://fields.scripps.edu/rawconv) and MZRefinery v.1.0 (https://omics.pnl.gov/software/mzrefinery) software, respectively, and then were searched by MS-GF+ algorithm v.9979 at 10 ppm precursor ion mass tolerance against the UniProt reference proteome database (55,152 entries, Mouse).
Data analysis	ImageJ v.1.52v and Image Lab v.6.0.1 were used to quantify western blot data. ZEN blue v.3.1 and Leica Application Suite X v.3.7.1.21655 were used to analyze imaging data. MS/MS spectrum annotation for biotinylated peptides was carried out using LcMsSpectator software v.1.1 (https://omics.pnl.gov/software/lcmsspectator).

For manuscripts utilizing custom algorithms or software that are central to the research but not yet described in published literature, software must be made available to editors and reviewers. We strongly encourage code deposition in a community repository (e.g. GitHub). See the Nature Research guidelines for submitting code & software for further information.

Data

Policy information about availability of data

All manuscripts must include a data availability statement. This statement should provide the following information, where applicable:

- Accession codes, unique identifiers, or web links for publicly available datasets
- A list of figures that have associated raw data
- A description of any restrictions on data availability

Proteomics data have been deposited to PRIDE server under accession code PXD025141 [https://www.ebi.ac.uk/pride/archive/projects/PXD025141]. Human liver

expression datasets were downloaded from the Human Protein Atlas (https://www.proteinatlas.org/). Signal peptides were predicted using the SignalP 5.0 sever (http://www.cbs.dtu.dk/services/SignalP/). Gene Ontology analysis was performed on a website powered by PANTHER (http://geneontology.org/). Protein species were retrieved by UniProt Retrieve/ID mapping (https://www.uniprot.org/uploadlists/). The raw data generated in this study are provided as a Source Data file. Source data are provided with this paper. Raw images of western blots and full sequence of plasmids used in this study are provided in the Source Data and Supplementary Data. All data that support the findings of this study are available from the corresponding author upon reasonable request.

Field-specific reporting

Please select the one below that is the best fit for your research. If you are not sure, read the appropriate sections before making your selection.

- [x] Life sciences
- [ ] Behavioural & social sciences
- [ ] Ecological, evolutionary & environmental sciences

For a reference copy of the document with all sections, see nature.com/documents/nr-reporting-summary-flat.pdf

Life sciences study design

All studies must disclose on these points even when the disclosure is negative.

Sample size	No statistical methods were used to predetermine sample sizes. Sample size was chosen based on the significance and consistency of differences between groups. n=3 is sufficient for this study because there was no biotinylated proteins in control mouse plasma.
Data exclusions	No data were excluded from the analysis.
Replication	Experiments were repeated more than once and all findings were reproduced.
Randomization	All mouse experiments were randomized for each group assignment.
Blinding	All experiments were not blinded. Blinding was not performed because we had to enrich biotinylated proteins only by streptavidin beads.

Reporting for specific materials, systems and methods

We require information from authors about some types of materials, experimental systems and methods used in many studies. Here, indicate whether each material, system or method listed is relevant to your study. If you are not sure if a list item applies to your research, read the appropriate section before selecting a response.

Materials & experimental systems

n/a	Involved in the study	
[ ]	[x]	Antibodies
[ ]	[x]	Eukaryotic cell lines
[x]	[ ]	Palaeontology and archaeology
[ ]	[x]	Animals and other organisms
[x]	[ ]	Human research participants
[x]	[ ]	Clinical data
[x]	[ ]	Dual use research of concern

Methods

n/a	Involved in the study	
[x]	[ ]	ChIP-seq
[x]	[ ]	Flow cytometry
[x]	[ ]	MRI-based neuroimaging

Antibodies

Antibodies used	Anti-GAPDH (CST, 2118), Anti-V5 (Invitrogen, R960-25), Cleaved Caspase-3 (CST, 9661), CHOP (CST, 2895), BiP (CST, 3177), Alexa Fluor goat anti-mouse immunoglobulin G (Invitrogen, A-11001), Alexa Fluor goat anti-Rabbit immunoglobulin G (Invitrogen, A-11010), Streptavidin-Alexa Fluor IgG (Invitrogen, S21374), Horse Anti-Mouse IgG Antibody (H+L) Peroxidase (Vector, PI-2000), Goat Anti-Rabbit IgG Antibody (H+L) Peroxidase (Vector, PI-1000), Streptavidin-HRP (Thermo, 21126)
Validation	Anti-GAPDH (CST, 2118) : https://www.cellsignal.com/products/primary-antibodies/gapdh-14c10-rabbit-mab/2118?Ntk=Products&Ntt=2118, Anti-V5 (Invitrogen, R960-25) : https://www.thermofisher.com/antibody/product/V5-Tag-Antibody-Monoclonal/R960-25, Cleaved Caspase-3 (CST, 9661) : https://www.cellsignal.com/products/primary-antibodies/cleaved-caspase-3-asp175-antibody/9661, CHOP (CST, 2895) : https://www.cellsignal.com/products/primary-antibodies/chop-l63f7-mouse-mab/2895?_=1613549450240&Ntt=2895&tahead=true, BiP (CST, 3177) : https://www.cellsignal.com/products/primary-antibodies/bip-c50b12-rabbit-mab/3177?_=1613549472063&Ntt=3177&tahead=true

Eukaryotic cell lines

Policy information about cell lines

Cell line source(s)	NIH-3T3, HepG2, AML12 and HeLa cell lines were purchased from the American Type Culture Collection (ATCC). 293AD cell line was purchased from the Cell Biolabs. Flp-In T-REx 293 cell line was purchased from the Thermo Fisher Scientific.

Authentication	The cell lines were not authenticated.
Mycoplasma contamination	The cell lines were not tested for mycoplasma contamination.
Commonly misidentified lines (See ICLAC register)	None.

Animals and other organisms

Policy information about studies involving animals; ARRIVE guidelines recommended for reporting animal research

Laboratory animals	All animal experiments were approved by the KAIST Institutional Animal Care and Use Committee (KAIST IACUC). 10-week-old C57BL/6J (JAX, 000664) male mice were used for all animal experiments. Mice were maintained under a 12 h light-dark cycle in a climate-controlled (23℃, 50% humidity), specific pathogen-free facility within the KAIST Laboratory Animal Resource Center. Standard chow diet (Envigo, 2018S) and water were provided ad libitum.
Wild animals	None.
Field-collected samples	None.
Ethics oversight	All animal experiments were approved by the KAIST Institutional Animal Care and Use Committee.

Note that full information on the approval of the study protocol must also be provided in the manuscript.
